# Understanding career choices of young medical professionals in Bangladesh: a mixed-methods study

**DOI:** 10.3389/fmed.2026.1925108

**Published:** 2026-09-16

**Authors:** Prapti Patricia Hagidok, Md Nuruzzaman, Taslima Chowdhury, Khandakar Fatema, M. Atiqul Haque

**Affiliations:** 1 Department of Public Health and Informatics, Bangladesh Medical University, Dhaka, Bangladesh; 2 World Health Organization, Dhaka, Bangladesh; 3 Department of Social and Cultural Sciences, Centre for Societal Risk Research (CSR), Karlstad University, Karlstad, Sweden

**Keywords:** Bangladesh, career choice, health workforce, medical education, physician migration, postgraduate training, workforce planning, young doctors

## Abstract

The health workforce in Bangladesh is undergoing demographic and structural changes, including increasing feminization and imbalances in specialty distribution. Understanding career choices among young doctors is important for workforce planning. A concurrent mixed-methods study was conducted in 2023 among 159 young medical professionals. Quantitative data were analysed using descriptive and inferential statistics, whereas qualitative data from interviews were analysed thematically. The findings from both components were integrated through triangulation, and an ecological model of career progression was developed to synthesize multilevel influences. Participants were mostly female (59.7%) with a mean age of 26.1 years. Almost all (99.4%) intended postgraduate training, mainly in clinical fields (93.0%), with medicine (53.5%) and surgery (34.0%) most preferred. Government service was the top career choice (65.4%). A large proportion also expressed interest in studying abroad (79.9%), and 28.3% planned permanent migration. Career decisions were influenced by work–life balance (91.2%), mental satisfaction (85.5%), personal interest (84.3%), and family responsibilities (76.7%). Qualitative findings revealed that career progression is often unplanned and delayed due to limited guidance, restricted exposure to non-clinical careers, and early career uncertainty. Structural barriers included lengthy recruitment processes, limited postgraduate training opportunities, inappropriate workplace conditions, and restricted career advancement in both the public and private sectors. Overall, career progression reflects interacting influences at individual, social, training, institutional, and policy levels, with global opportunities serving as an alternative pathway amid perceived domestic constraints. Career progression among young doctors in Bangladesh is influenced by both individual motivations and structural constraints. Strengthening career guidance, expanding training opportunities, and improving working conditions are essential for building a balanced and sustainable health workforce.

## 1 Introduction

The global health workforce faces persistent challenges related to distribution, retention, and professional satisfaction, particularly in low- and middle-income countries (LMICs) (1). These challenges extend beyond workforce shortages to include imbalances in specialty distribution and geographic deployment, often resulting in inadequate access to healthcare services in underserved areas and a compromised quality of care (2).

Meanwhile, the medical workforce is becoming increasingly feminized, with a growing proportion of female medical students and practitioners. This shift has important implications for specialty preferences, work patterns, and service delivery (3).

Career choice is a critical determinant of the professional trajectory in medicine, where individual aspirations intersect with societal needs. Decisions related to entry into the profession and subsequent specialty selection are shaped by a combination of intrinsic and extrinsic factors, including personal interests, social prestige, financial expectations, and family influence (4, 5). Understanding these drivers is essential for workforce planning, curriculum design, and ensuring equitable access to healthcare services (6).

Globally, most medical students demonstrate a strong preference for clinical specialties over non-clinical fields, such as basic sciences and public health (7). Bangladesh reflects a similar pattern. Previous studies have shown that male students tend to prefer surgical specialties, whereas female students are more inclined toward medical specialties, with personal interest and employment opportunities acting as key determinants (6). However, such preferences contribute to persistent workforce imbalances, particularly the underrepresentation of preventive and public health disciplines (8), which may hinder broader population health outcomes.

Motivation theory explains why a physician is attracted to, persists in or withdraws from a specialty. Career motivations are not static; they evolve over time. Early career decisions are often driven by academic interests and professional aspirations, whereas later choices are shaped by practical considerations such as income, job security, and family responsibilities (9). In contexts such as Bangladesh, these evolving priorities influence both specialty choice and workplace preferences.

Self-Determination Theory (4) distinguishes between intrinsic motivation, engagement driven by personal interest and satisfaction, and extrinsic motivation, shaped by external rewards or pressures such as social prestige, financial security, or family expectation. This framework underpins how career decisions in medicine are understood: decisions related to entry into the profession and subsequent specialty selection are shaped by a combination of intrinsic and extrinsic factors, including personal interests, social prestige, financial expectations, and family influence (4, 5). Systems theory explains how that motivation is shaped through interactions among the individual and nested social, educational, organizational and societal environments. Systems theory extends the motivational account by locating the physician within a set of interacting environments. An expanded framework for primary-care career choice applies four nested systems-microsystem, mesosystem, exosystem and macrosystem- to represent the multiple levels that influence medical students throughout their education (10).

Physician career choice should not be conceptualized as a one-time individual preference or as the direct result of a single motive. It is a developmental and context-dependent process in which students and physicians interpret their interests, abilities, identities, relationships, training experiences, labor-market conditions and professional expectations. A systematic review of medical career-choice research classified relevant influences into student characteristics, personal values, career needs such as income and work-life balance and perceptions of specialty characteristics; the review concluded that career decisions often emerge from a match between perceived specialty attributes and personal needs (11).

Institutional and systemic structures further shape career pathways. Institutional theory suggests that physician career choices are shaped by the institutional environments in which medical education and practice occur. These environments establish professional expectations, organize rewards and status and expose physicians to competing institutional logics, including professional service and market-oriented pressures. In medical career-choice research, medical-school characteristics, specialty status, expected income, work-life balance and perceptions of specialty practice are associated with how students evaluate available career options (11). The Bangladesh Public Service Commission plays a central role in recruiting physicians into government services, while the Ministry of Health and Family Welfare (MOHFW), through the Directorate General of Health Services (DGHS), oversees service delivery (12, 13). Although government services are often associated with stability and social prestige, factors such as bureaucratic delays, limited training opportunities, and suboptimal working conditions may influence career decisions. These constraints are not unique to individual institutions but reflect broader gaps in physician-specific workforce planning and supervision structures within the health system (2, 11), which may help explain why structural barriers persist despite the continued preference for government service.

The transition from being a medical student to a practicing physician presents additional challenges. Many students from Bengali-medium backgrounds face difficulties adapting to English-language medical curricula, which can affect learning and confidence. At the same time, intense competition for postgraduate training, limited training seats, and the absence of structured career guidance create uncertainty during early career stages (9).

Despite existing evidence, there is a lack of comprehensive mixed-methods research that captures both the patterns of career choices and the underlying contextual and experiential factors shaping these decisions in Bangladesh. Most studies have focused on specialty preferences without adequately exploring broader systemic and personal influences.

Therefore, this study aimed to examine the career choices and influencing factors among young medical professionals in Bangladesh. Specifically, it explored their career aspirations, identified determinants of career planning, examined challenges in career progression, and assessed perceived opportunities for advancement. The quantitative component addressed the first two objectives by quantifying the pattern and prevalence of career aspirations and the factors motivating them, while the qualitative component addressed the latter two by capturing the lived experiences underlying career progression, including the barriers encountered and the opportunities perceived within the system. The findings are intended to inform policies that support a balanced, responsive, and sustainable health workforce.

## 2 Materials and methods

### 2.1 Study design, setting and population

This study adopted a concurrent mixed-methods design, integrating quantitative and qualitative approaches to comprehensively explore career choices and influencing factors. The quantitative component was a facility-based cross-sectional survey, whereas the qualitative component included in-depth interviews (IDIs) and Key Informant Interviews (KIIs). Both components were conducted simultaneously and integrated through triangulation to enhance validity and the depth of interpretation. Given that the study aimed to both document the pattern of career preferences and examine the underlying determinants and challenges shaping them, the quantitative component was designed to establish the distribution and prevalence of career choices, motivations, and preferences across the sample, while the qualitative component was designed to explain the mechanisms and contextual factors underlying these patterns**, reflecting** a complementarity rather than a corroboration function of triangulation.

This study was conducted between January and December 2023 at selected healthcare facilities in the Dhaka division of Bangladesh. Eight Upazila Health Complexes (UHCs) were randomly selected from a total of 38 government UHCs (50-bed facilities) using SPSS-generated random numbers. The selected UHCs were located in Kashiani (Gopalganj), Gazaria (Munshiganj), Bhuapur, Sakhipur and Gopalpur (Tangail), Kaliganj (Gazipur), Shibchar (Madaripur), and Bhanga (Faridpur).

To capture the variation across healthcare settings, four medical college hospitals were purposively included: two government institutions (Mymensingh Medical College Hospital and Sir Salimullah Medical College Mitford Hospital) and two private institutions (Bangladesh Medical College Hospital and Dr. Sirajul Islam Medical College Hospital).

The study population comprised early-career medical professionals, including medical officers appointed to Upazila Health Complexes through the Bangladesh Civil Service (BCS) (Health cadre) and intern doctors working in selected medical college hospitals. Medical officers from the 39th and 42nd BCS batches were included, representing physicians at a critical stage of career decision-making. Intern doctors were included to capture perspectives during the transition from students to practicing professionals.

### 2.2 Sample size and sampling strategy

For the quantitative component, the required sample size was calculated as 147, assuming a 75% prevalence of career preference, a 95% confidence level, and a 7% margin of error (6). Ultimately, 159 participants were included in this study. Eligible participants present during the data collection period were recruited using convenience sampling.

Eligible intern doctors and medical officers were recruited during routine duty hours because clinical service schedules limited the feasibility of probability sampling. This approach enabled efficient recruitment of participants at an important career stage while maintaining diversity across government and private facilities.

For the qualitative component, 15 participants were recruited, including BCS doctors, intern doctors, and key informants such as administrators and senior physicians. A purposive sampling approach ensured diversity in roles and experiences. Fifteen interviews were conducted, including 12 in-depth interviews and three key informant interviews. The participants included seven doctors from Upazila Health Complexes, five intern doctors from medical college hospitals, two Upazila Health and Family Planning Officers, and one deputy program manager.

Participants for interviews were purposively selected to ensure variation in professional roles, career stages, institutional settings and administrative responsibilities, thereby capturing a broad range of experiences relevant to career progression.

### 2.3 Data collection tools, procedures and measurements

Quantitative data were collected using a semi-structured, self-administered questionnaire developed based on relevant literature and pre-tested prior to use. The tool included sections on sociodemographic characteristics, motivations for choosing medicine, specialty preferences, and influencing factors. Institutional permission was obtained prior to data collection. Participants were approached during duty hours, provided with study information, and asked to provide written informed consent before completing the questionnaire. For the qualitative component, semi-structured interview guides were used. Interviews explored career aspirations, motivations, challenges, and opportunities. All interviews were conducted in Bengali, audio-recorded with consent, and transcribed verbatim.

The primary outcome variables were postgraduate specialty choice and motivations for choosing a medical career. Specialty preferences were categorized as medicine, surgery, obstetrics and gynaecology, paediatrics, and non-clinical fields.

The explanatory variables included sociodemographic characteristics, professional attributes, and the perceived importance of influencing factors. Factors influencing work satisfaction, such as work-life balance, mental satisfaction, income, and family responsibilities, were measured using Likert-type scales.

### 2.4 Data management and analysis

Data were checked for completeness and consistency before entry and analyzed using SPSS version 26. Descriptive statistics were used to summarize participant characteristics. Associations between categorical variables were assessed using chi-square tests, with statistical significance set at *P* < 0.05. Qualitative data were analyzed using thematic analysis. Transcripts were iteratively coded, and codes were grouped into broader themes aligned with the study objectives. Data integration followed a convergent triangulation design: quantitative findings describing the prevalence and distribution of career preferences and influencing factors were compared side by side with qualitative themes exploring participants' experiences and explanations, construct by construct (e.g., specialty preference, sector preference, motivators, and barriers), to identify areas of convergence, divergence, and complementarity. Where quantitative findings established how common a preference or barrier was, qualitative findings were examined for the reasoning or mechanism underlying it. The resulting synthesis was organized using an ecological model of career progression, structured by level of influence, individual, family/social, training, institutional, and policy reflecting the multilevel pattern that emerged when the two data strands were compared. Reporting follows the STROBE statement for the cross-sectional component and the COREQ checklist for the qualitative component.

### 2.5 Ethical considerations

Ethical approval was obtained from the Institutional Review Board (IRB) of Bangladesh Medical University (BMU), Memo no. BSMMU/2023/10679. Participation was voluntary, and written informed consent was obtained from all participants. Confidentiality was ensured through anonymization and secure data handling. No identifiable personal information was included in the analysis or reporting, and the study involved minimal risk with no invasive procedures.

## 3 Results

### 3.1 Quantitative findings

#### 3.1.1 Sociodemographic and professional profile

Of the 159 respondents, 59.7% were female. The mean (SD) age was 26.08 (1.60) years. Most participants were intern doctors (78.6%), while 21.4% were medical officers. More than two-thirds (67.9%) reported working more than 12 h per day, and 10.1% practiced in private chambers despite their hospital duties.

Regarding personal characteristics, 72.3% were unmarried and 91.8% had no children. Most respondents reported that their mothers were housewives (83.6%) and their fathers were service holders (76.7%) (Table 1).

**Table 1:** Background and sociodemographic information (*n* = 159).

Variables	Total, *n* (%)
Sex	
Male	64 (40.3)
Female	95 (59.7)
Age, mean (SD), year	26.08 (1.60)
Residence	
Urban	137 (86.2)
Rural	22 (13.8)
Mother's profession	
Housewife	133 (83.6)
Other	26 (16.4)
Father's profession	
Service holder	122 (76.7)
Other	37 (23.3)
Current position	
Medical officer	34 (21.4)
Intern doctor	125 (78.6)
Hospital working hour	
8–12 h	51 (32.1)
>12 h	108 (67.9)
Engaged in private chamber practice	16 (10.1)
Marital status	
Unmarried	115 (72.3)
Married	44 (27.7)
Having children	**13** (**8.2)**

#### 3.1.2 Career planning and preferences

Almost all respondents (99.4%) expressed an interest in pursuing postgraduate training. A large majority preferred clinical disciplines (93.0%), 5.1% preferred non-clinical fields, and 1.9% reported mixed preferences (Table 2).

**Table 2:** Career planning among young doctors (*n* = 159).

Domain and category	Male	Female	Total
Interested in post-graduation	64 (100.0)	94 (98.9)	158 (99.4)
Clinical	60 (93.8)	87 (92.6)	147 (93.0)
Nonclinical	3 (4.7)	5 (5.3)	8 (5.1)
Mixed	1 (1.6)	2 (2.1)	3 (1.9)
Preferred work sector^a^			
Government	43 (67.2)	61 (64.2)	104 (65.4)
Non-government/private	5 (7.8)	9 (9.5)	14 (8.8)
Abroad	19 (29.7)	30 (31.6)	49 (30.8)
Independent practice	11 (17.2)	5 (5.3)	16 (10.1)
Preferred career pathway			
Admin	2 (3.1)	3 (3.2)	5 (3.1)
Clinical	61 (95.3)	89 (93.7)	150 (94.3)
Teacher	9 (14.1)	11 (11.6)	20 (12.6)
Career intentions (international)^a^			
Intention for higher study abroad	52 (81.3)	75 (78.9)	127 (79.9)
Intention for permanent settlement abroad (*n* = 159)			
Yes	14 (21.9)	31 (32.6)	45 (28.3)
No	34 (53.1)	39 (41.1)	73 (45.9)
Not decided yet	16 (25.0)	25 (26.3)	41 (25.8)

Data are presented as numbers (percentages).

a Multiple responses were allowed; therefore, the percentages may exceed 100%.

Medicine (53.5%) and surgery (34.0%) were the most commonly preferred specialties, followed by paediatrics (16.4%) and obstetrics and gynaecology (13.8%). The preference for non-clinical fields, including basic sciences and public health, was low (5.1%; Table 2).

Government services were the most commonly preferred employment sector (65.4%), followed by working abroad (30.8%), independent practice (10.1%), and the private/non-government sector (8.8%).

Most respondents preferred a clinical career pathway (94.3%), while smaller proportions reported an interest in teaching (12.6%) and administrative roles (3.1%).

Most (79.9%) expressed an intention to pursue higher studies abroad, while 28.3% intended to establish a permanent career abroad and 25.8% remained undecided.

#### 3.1.3 Faculty-wise distribution of specialty preferences

When classified by faculty, medicine and allied disciplines accounted for 62.9% of preferences, followed by surgery and allied fields (37.7%) and paediatrics (15.1%) (Table 3).

**Table 3:** Faculty-wise distribution of preferred subject areas (*n* = 159).

Faculty	Subject areas included	n (%)^a^^,^^b^
Medicine and allied	Medicine, Cardiology, Endocrinology, Gastroenterology, Hepatology, Nephrology, Neurology, Rheumatology, Respiratory medicine, Critical care, Dermatology, Psychiatry	100 (62.9)
Surgery and allied	Surgery, Orthopaedics, Ophthalmology, ENT, Radiology, Obstetrics & gynaecology, Anaesthesiology	60 (37.7)
Paediatrics and allied	Paediatrics, Neonatology	24 (15.1)
Faculty of Preventive and Social Sciences	Public health	3 (1.9)
Basic and paraclinical sciences	Pathology, Microbiology, Pharmacology, Biochemistry, Anatomy, Physiology	11 (6.9)

Percentages are calculated using the total sample (n = 159).

a Multiple responses were allowed; therefore, the percentages exceed 100%.

b Some respondents categorized under clinical and non-clinical groups did not specify any particular subject preference.

Public health under the Faculty of Preventive and Social Sciences accounted for 1.9% of the responses, while basic and paraclinical sciences accounted for 6.9%.

A small number of respondents did not specify a particular subject preference despite indicating a clinical (*n* = 11) or non-clinical (*n* = 3) orientation.

#### 3.1.4 Motivation for postgraduate training and rural service

Personal interest was the most frequently reported motivator for pursuing postgraduate education (84.3%), followed by family influence (29.6%), and influence from senior doctors (27.0%). Peer influence was less commonly reported (5.7%) (Table 4).

**Table 4:** Motivation for pursuing postgraduate degree by sex (*n* = 159).

Variable	Total, n (%)	Male, n (%)	Female, n (%)	*P* value
Motivators for post-graduation				
Personal interest	134 (84.3)	52 (81.3)	82 (86.3)	.405
Family members	47 (29.6)	15 (23.4)	32 (33.7)	.195
From peers	9 (5.7)	0 (0.0)	9 (9.5)	.011
From a senior doctor	43 (27.0)	24 (37.5)	19 (20.0)	.017
Others	2 (1.3)	2 (3.1)	0 (0.0)	.085
Interest in serving rural area				
Not interested	20 (12.6)	6 (9.4)	14 (14.7)	
Want to serve for some time	116 (73.0)	51 (79.7)	65 (68.4)	.292
Want to serve for a long time	23 (14.5)	7 (10.9)	16 (16.8)	

Percentages were calculated within sex categories.

Multiple responses were allowed; therefore, the percentages may exceed 100%.

A sex-disaggregated analysis showed that peer influence was reported only among female respondents (9.5% vs. 0.0%, *p* = 0.011), whereas influence from senior doctors was more common among male respondents (37.5% vs. 20.0%, *p* = 0.017).

Regarding rural service, 73.0% of respondents reported willingness to serve in rural areas for some time, and 14.5% for a longer duration. No statistically significant sex differences were observed.

#### 3.1.5 Factors influencing specialty choice

Respondents rated multiple factors influencing specialty choice. Work–life balance was the most frequently rated as extremely important (91.2%), followed by mental satisfaction (85.5%) and family responsibilities (76.7%).

Other factors rated as extremely important included required aptitude (72.3%), transport access (72.3%), and workplace location (65.4%). Career prospects (58.5%) and potential earnings (56.0%) were also frequently reported.

The spouse's occupation was rated as extremely important by 38.4% of the respondents. Patient-related factors, including patient load and continuity, were less frequently rated as extremely important.

Figure 1 presents the mean scores with 95% confidence intervals. Lower mean scores indicate higher importance. Work–life balance (mean 1.09, 95% CI 1.04–1.14), mental satisfaction (1.16, 95% CI 1.10–1.22), and family responsibilities (1.25, 95% CI 1.19–1.31) had the lowest mean scores. In contrast, patient continuity (2.12, 95% CI 1.98–2.26) and patient load (1.89, 95% CI 1.77–2.01) had higher mean scores.

**Figure 1:**
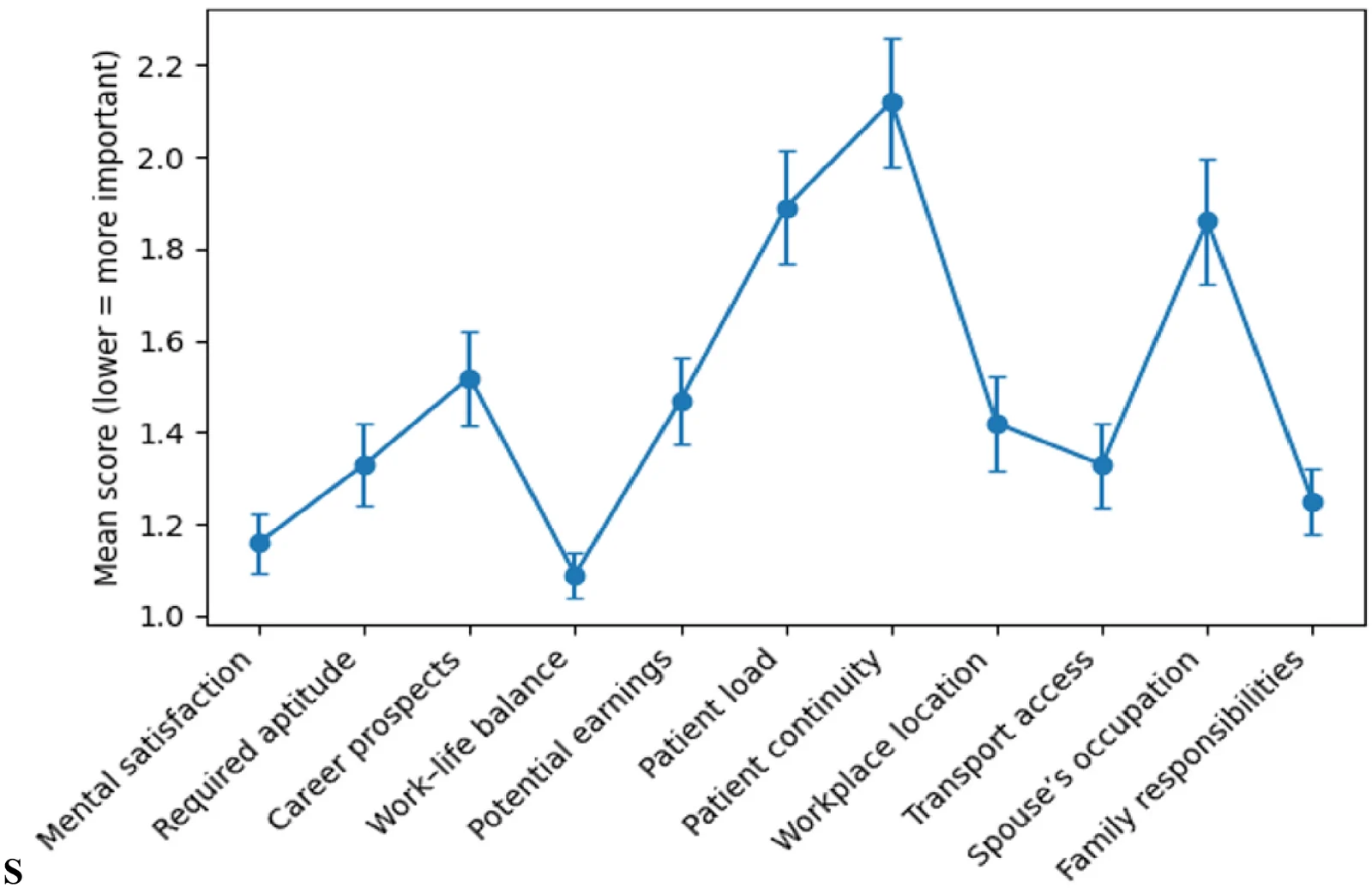
Factors influencing career choice (mean with 95% confidence interval).

### 3.2 Qualitative findings

The thematic analysis identified five interrelated themes shaping career progression among young doctors: (1) early career orientation and aspirations, (2) motivation to enter the profession, (3) structural and professional barriers to career progression, (4) system-level gaps and recommendations, and (5) perceived opportunities within the career pathway.

#### 3.2.1 Early career orientation and aspirations

Career progression challenges appeared to originate early at the stage of career orientation. Many participants reported difficulty making timely and informed decisions about specialization, largely due to the absence of structured mentorship and career guidance.

“No, I haven't decided yet. First of all, I want to complete my internship in all the departments.” [IDI Intern-03]

This delay in decision-making often results in missed opportunities for early preparation in postgraduate pathways.

“If I could have decided a little earlier, then I could have started doing the MPH earlier… we don't really get the guidelines that way.” [KII 03]

Career aspirations remain heavily skewed toward a limited number of traditional clinical specialties, reflecting a narrow understanding of career pathways and limited exposure to alternative roles.

“I still don't see people saying they want to be epidemiologists or public health specialists… everyone is still thinking in the same old way.” [KII 02]

In terms of long-term planning, government employment was widely perceived as the most stable career trajectory, while international migration was viewed as an alternative pathway to better professional growth and a higher standard of living.

“If I stay in the country… a government job is the best for me.” [IDI 02]

“I have a general desire to do postgraduate studies and then build a career abroad…” [IDI Intern 04]

#### 3.2.2 Motivation for entering the profession

Motivational factors also shape career progression trajectories. Family expectations and social prestige often influence the decision to enter medicine, sometimes without a clear long-term career plan.

“There is also the desire of the family… parents also have a desire that one of their children should be a doctor.” [KII 03]

Exposure to family members in the same profession further reinforces career choices.

“My father is a cardiologist… seeing him, I have a kind of interest for the profession.” [IDI 06]

Simultaneously, intrinsic interest, particularly in biology, was a key driver for many participants.

“I have been interested in biology since the intermediate level…” [IDI Intern 03]

However, these motivations are often not accompanied by structured career planning, contributing to later uncertainty and delayed progression.

#### 3.2.3 Structural and professional barriers to career progression

##### 3.2.3.1 Government sector barriers

Although government services were considered the most desirable pathway, multiple systemic barriers hindered career progression. A major concern was the lack of structured training and orientation for the newly recruited doctors, particularly in terms of managerial and administrative responsibilities.

“From the day we join, we are doing emergency duty… but there is no supervision.” [KII 03]

Participants highlighted that unlike other civil service cadres, doctors enter service without adequate preparation, which affects both performance and confidence.

The prolonged and bureaucratic BCS recruitment process further delays entry into stable career paths.

“It takes people two, three or four years to complete this process.” [KII 02]

Additionally, limited promotion opportunities, high competition, and a lack of supportive working conditions were reported to impede long-term career growth.

#### 3.2.4 Private sector barriers

The private sector was not perceived as a viable early-career pathway because of low remuneration, lack of job security, and limited opportunities for professional advancement.

“The salaries are not good and there is not much opportunity for research.” [IDI Intern 02]

“Unless you are a specialist… there are very few opportunities… dissatisfaction about the salary scale.” [KII 03]

This limited attractiveness of the private sector further constrains career options and increases pressure on government job pathways.

#### 3.2.5 Academic and training-related barriers

Career progression was also hindered by challenges within the undergraduate and postgraduate training systems. The MBBS curriculum is described as intensive and difficult, with additional challenges related to language barriers.

“There is a language barrier… and it is a very intensive and difficult course.” [KII 02]

Participants also highlighted the limited availability of postgraduate seats and difficulties in entering desired specialties.

“The number of seats in postgraduate and government jobs is very low…” [IDI Intern 03]

A shortage of qualified faculty in basic sciences further affects the quality of foundational training.

“There is a shortage of basic subject teachers in the country right now.” [KII 01]

#### 3.2.6 Societal and professional perception

Negative societal perceptions of doctors were identified as an additional barrier that potentially affected professional identity and motivation.

“People have a negative perception that doctors will take a percentage (commission) from everything (e.g., diagnostic test).” [KII 03]

#### 3.2.7 System-level gaps and recommendations for improving career progression

Participants consistently emphasized the need for systemic reforms to facilitate smoother career progression. Strengthening research capacity and aligning healthcare systems with evolving needs are considered essential.

“More research is needed… to create a good healthcare system.” [IDI 07]

The reform of posting policies, particularly ensuring flexibility and district-based placement, was suggested to improve retention and career satisfaction.

“If the rules are district-based… the upazilas and the periphery will never be empty.” [IDI 04]

Improving workplace conditions, including safety, infrastructure, and supportive supervision, was highlighted as a critical factor in sustaining career engagement.

“If I can provide good accommodation and transportation, doctors will definitely stay.” [KII 03]

The participants also stressed the need to regulate postgraduate admissions and improve the quality of medical education, particularly in the rapidly expanding private sector.

“There is a huge difference in quality… this will destroy this profession.” [KII 02]

#### 3.2.8 Opportunities within the career pathway

Despite these challenges, participants acknowledged certain structured opportunities within the system, particularly in the government sector. The possibility of pursuing postgraduate training after a defined period of service was identified as a key facilitator of career progression.

“After two years of compulsory service… you can do post-graduation.” [KII 03]

These opportunities are viewed as important incentives, although their effectiveness depends on equitable access and system efficiency.

#### 3.2.8 Integrated interpretation of quantitative and qualitative findings

The quantitative and qualitative findings were largely convergent and together illustrated a multilevel pathway of career progression among young doctors. Convergence was assessed by comparing each quantitative result against the corresponding qualitative theme; for example, the quantitative preference for government service (65.4%, Table 2) aligned with qualitative accounts of perceived job security, while the low interest in non-clinical specialties (5.1%, Table 2) corresponded to qualitative reports of limited exposure to non-clinical careers. To synthesize these findings, an ecological model of career progression was developed (Figure 2), demonstrating how individual preferences are shaped and constrained by influences at multiple levels.

**Figure 2:**
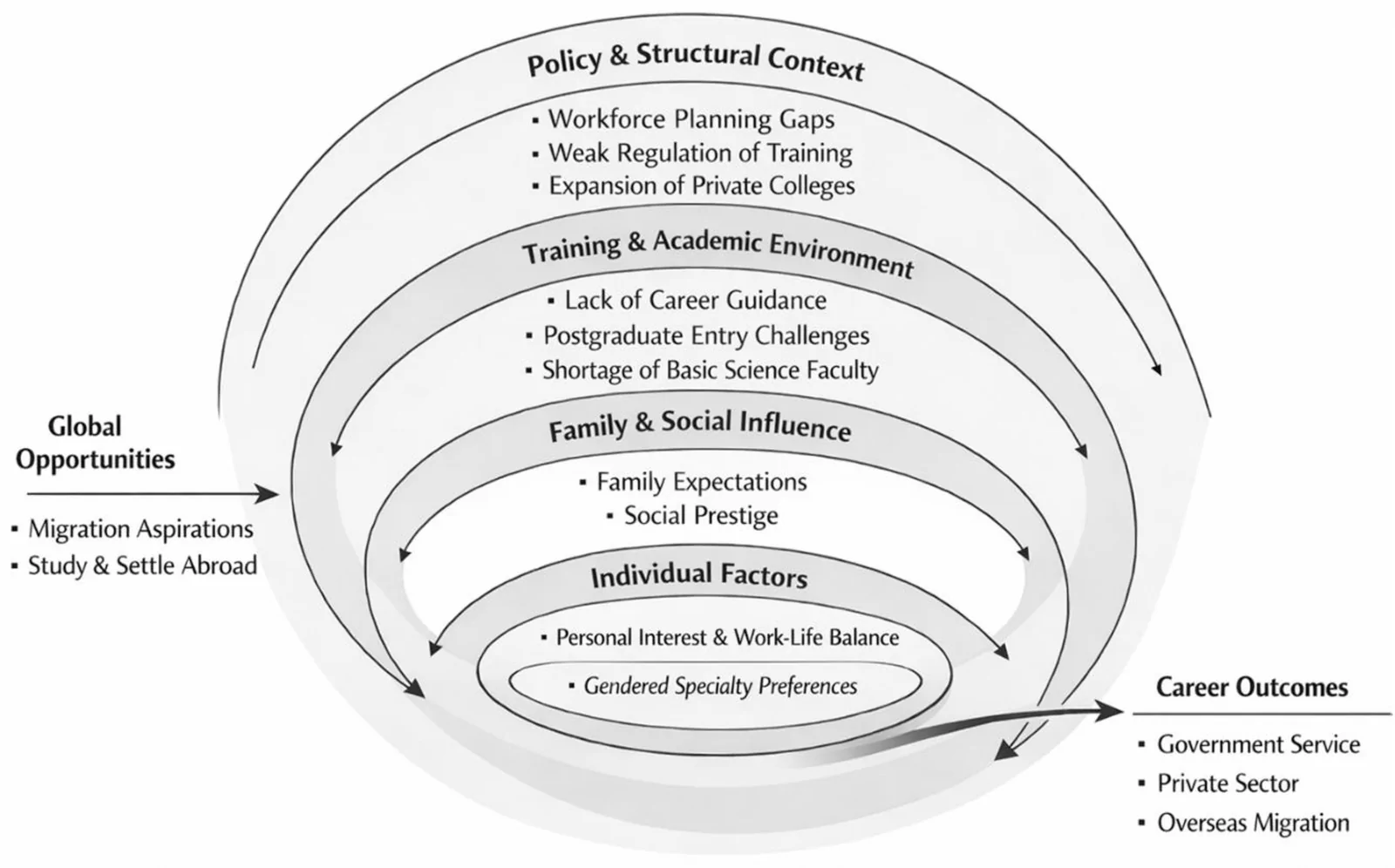
Ecological model of career progression among young doctors in Bangladesh.

At the individual level, career decisions were primarily driven by personal interest (84.3%), work–life balance, mental satisfaction, and family responsibilities. These factors represent the core drivers of early career orientation and specialty preference. However, qualitative findings showed that these preferences were often formed without structured planning, with many participants reporting delayed decision-making and uncertainty during internships.

At the family and social levels, family expectations and professional backgrounds influenced entry into medicine and reinforced socially accepted career pathways. This was reflected in both survey findings (family influence: 29.6%) and interview narratives describing parental expectations and role modelling within the profession.

At the level of training and academic environment, both data strands highlighted important constraints shaping career progression. Quantitative findings showed an overwhelming preference for clinical disciplines (93.0%), while qualitative findings revealed that this was linked to limited exposure to non-clinical careers, lack of mentorship, language barriers, and challenges in postgraduate entry. These factors contributed to the narrowing of career options early in the professional trajectory.

At the health system and institutional levels, structural barriers played a central role in shaping career pathways. The strong preference for government service (65.4%) identified in the survey was supported by qualitative findings describing perceived job security. However, interviews revealed that this pathway is constrained by bureaucratic delays in the BCS recruitment process, lack of structured induction and supervision, limited postgraduate training opportunities, and suboptimal working conditions. Although the private sector was available, it was not perceived as a viable alternative because of low salaries and limited career advancement opportunities.

At the policy and structural levels, systemic issues, such as workforce planning gaps, variations in the quality of medical education, and limited regulation of postgraduate training, further influenced career progression. These factors contributed to a mismatch between the high demand for specialization and the limited availability of training positions.

Finally, global opportunities emerged as a cross-cutting influence on career decisions. The high proportion of respondents expressing interest in higher studies abroad (79.9%) and permanent migration (28.3%) reflects a parallel career pathway shaped by perceived limitations within the domestic system. Qualitative findings further highlighted the role of better training environments and improved lifestyles in driving this aspiration.

Taken together, these findings suggest that career progression among young doctors in Bangladesh is shaped by interconnected influences at multiple levels. While individual motivation and preferences initiate the process, progression is significantly shaped and often constrained by training environments, institutional structures, and broader policy contexts. This results in a fragmented career pathway, with many doctors navigating between government services, limited domestic opportunities, and aspirations for international migration.

## 4 Discussion

This study provides a comprehensive understanding of career choices and progress of young medical professionals in Bangladesh. Consistent with their respective roles in the design, the quantitative findings established the extent and pattern of career aspirations and their motivators, while the qualitative findings explained the challenges and opportunities shaping career progression. The findings show a strong preference for clinical specialties, particularly medicine and surgery, along with a strong intention to pursue postgraduate training. However, career progression is shaped not only by individual motivation but also by structural and institutional constraints that influence decision-making at different stages.

### 4.1 Career choice and influencing factors

The study identified medicine, surgery, obstetrics and gynaecology, and paediatrics as the most preferred specialties. Specialty preferences were not analysed by sex at the level of individual disciplines in this sample; the wider literature, however, consistently reports that female physicians are more likely to prefer obstetrics and gynaecology and paediatrics. This aligns with existing literature suggesting that female physicians often choose specialties perceived as more compatible with family responsibilities and work–life balance (14, 15). Consistent with our findings, a recent study in Bangladesh reported a gradual increase in female physicians across specialties, particularly in surgery, public health, and basic sciences (16), indicating a shifting gender distribution within the medical workforce.

Interest in non-clinical disciplines, including public health and basic sciences, was notably low. Similar findings have been reported in Bangladesh and other LMICs, where preventive and social medicine remain underrepresented, despite their importance to population health (8). This imbalance reflects both limited exposure during training and the perceived lack of clear career pathways in non-clinical fields. The qualitative accounts locate one proximate cause in the shortage of basic-science faculty reported by senior respondents, which limits both exposure to and role modelling within these disciplines and so sustains the imbalance across successive cohorts.

Personal interest emerged as the most important motivator for choosing medicine and a specialty, consistent with global evidence (17). This predominance of intrinsic over extrinsic motivation is consistent with Self-Determination Theory (4), which holds that intrinsically motivated choices tend to be associated with greater sustained engagement and satisfaction than those driven primarily by external pressures. Other key motivators included the desire to serve patients and the social prestige associated with the profession. These findings are in line with studies from Sierra Leone and the UK, where altruism, family influence, and societal expectations play a central role in career choice (18, 19).

Sex differences were evident in the reported sources of motivation for postgraduate training. Influence from a senior doctor was reported more often by male respondents (37.5% versus 20.0%, *p* = 0.017), whereas peer influence was reported only by female respondents (9.5% versus 0.0%, *p* = 0.011) (Table 4). Motivations relating to income and lifestyle were not analysed by sex and are therefore not compared here. This reflects broader sociocultural expectations and highlights the need for gender-sensitive workforce planning.

### 4.2 Career progression and opportunities

A key finding of this study is the near-universal intention to pursue postgraduate training, primarily in the clinical field. This reflects the perception that specialization is essential for career advancement in Bangladesh. However, qualitative findings suggest that career progression is often unstructured and delayed, with many doctors lacking clear guidance in the early stages of their careers.

Government service was the most preferred employment pathway, largely because of perceived job security. This finding aligns with those from other settings, where public sector employment is favored despite lower financial incentives (20). At the same time, a substantial proportion of participants expressed interest in studying and working abroad, indicating the presence of parallel career trajectories influenced by perceived limitations within the domestic system.

The limited awareness of career opportunities and pathways among participants suggests gaps in career counselling and workforce planning. This lack of clarity may contribute to delayed decision-making and inefficient career progression.

### 4.3 Influencing factors for specialty choice

Work–life balance emerged as the most important factor influencing specialty choice, followed by mental satisfaction and family responsibilities. These findings are consistent with global evidence that lifestyle considerations increasingly shape specialty preferences (14).

Other factors, including aptitude, workplace location, and career prospects, were also important, although to a lesser extent. In contrast, patient-related factors, such as workload and continuity of care, were less influential, suggesting a shift toward lifestyle-oriented decision-making among younger doctors.

These findings indicate that specialty choice is no longer driven solely by academic interest or prestige but increasingly reflects broader life priorities. This has important implications for workforce distribution, particularly in high-demand but demanding specialties.

### 4.4 Challenges in career progression

The qualitative findings highlight several structural barriers affecting career progression. First, the transition from medical education to professional practice is challenging because of the intensive curriculum, language barriers, and limited faculty support. Similar challenges have been reported in other LMIC contexts, where educational systems may not adequately prepare graduates for clinical and administrative roles.

Second, limited availability of postgraduate training seats and competitive admission processes create significant bottlenecks in career advancement. This mismatch between the high demand for specialization and limited training capacity is a critical issue for workforce planning.

Third, systemic barriers within the health system, including lengthy recruitment processes, lack of structured training for newly recruited doctors, and inadequate working conditions, further constrain career progression. These findings highlight the gap between policy intentions and implementation realities.

The private sector was not perceived as a viable alternative pathway because of low salaries, limited job security, and restricted opportunities for professional development. Additionally, negative societal perceptions of doctors were reported, which may affect professional satisfaction and motivation. Participants attributed this perception to commission-based referral practices associated with private diagnostic services rather than to clinical care itself, indicating that the reputational cost is borne by the profession as a whole while the incentive operates at the level of the private market.

Viewed through the lens of Self-Determination Theory (4), the structural barriers described above (delayed recruitment, absent supervision, and restricted training capacity) represent conditions that constrain autonomy and competence even where intrinsic motivation is high, offering a theoretical account of why strong personal interest in medicine did not translate into a clear or unimpeded career pathway. Overall, these challenges contribute to a fragmented career pathway, in which progression is often delayed and uncertain.

### 4.5 Implications for policy and practice

The findings suggest that career progression among young doctors in Bangladesh is shaped by interacting factors at individual, institutional, and systemic levels. The ecological model developed in this study illustrates how these multilevel influences create both opportunities and constraints.

Policy interventions should focus on the following:

1. Strengthening structured career guidance and mentorship during undergraduate and early professional stages
2. Expanding postgraduate training capacity and ensuring equitable access
3. Improving working conditions and support systems, particularly in rural and peripheral settings
4. Promoting non-clinical career pathways, including public health and health administration
5. Addressing systemic inefficiencies in recruitment and training processes

Each recommendation traces to a specific point of convergence between the two data strands rather than to either strand alone. The near-universal intention to pursue postgraduate training (99.4%, Table 2) converged with qualitative reports of limited and competitive training seats to support recommendation 2, while the high proportion willing to serve in rural areas at least temporarily (73.0%, Table 4) converged with qualitative accounts identifying accommodation and transport as conditions for retention to support recommendation 3. Recommendations arising from a single data strand alone, for instance, the quantitative pattern without qualitative explanation, or vice versa, were treated as preliminary observations rather than a basis for policy direction, which is why some barriers identified in only one component (e.g., the sex-disaggregated differences in senior-doctor and peer influence, Table 4) are discussed as findings but do not appear as standalone recommendations. Such measures are essential for ensuring a balanced, responsive, and sustainable health workforce.

### 4.6 Limitations of the study

This study has several limitations. First, the findings may not be generalizable to the entire country, as data were collected from a limited number of facilities within one administrative division. Second, the cross-sectional design limits the ability to establish causal relationships between variables. A longitudinal approach would provide a better understanding of how career preferences evolve over time. Third, self-reported data may be subject to recall and social desirability biases. Fourth, participants were recruited by convenience during duty hours, which may over-represent doctors present on the days of data collection and limits the precision of the prevalence estimates reported. Fifth, thematic saturation was not formally documented for the qualitative component, so the themes should be read as indicative of the range of experience rather than exhaustive.

## 5 Conclusion

This study demonstrates that career choices among young medical professionals in Bangladesh are driven by strong individual motivation, particularly personal interest, consistent with Self-Determination Theory (4), but are significantly shaped by structural and systemic factors. There is a marked preference for clinical specialties and government employment, alongside a growing inclination toward international training and migration. Career progression is often unstructured and delayed due to limited career guidance, restricted postgraduate training opportunities, and institutional barriers such as lengthy recruitment processes and suboptimal working conditions. At the same time, the low interest in non-clinical fields highlights an imbalance that may affect the broader health system, particularly in public health and preventive services.

Taken together, these findings indicate that career progression follows a fragmented pathway influenced by interacting factors at individual, social, and system levels. Addressing these challenges requires coordinated policy actions, including strengthening career guidance and mentorship, expanding postgraduate training capacity, improving working environments, and promoting diverse career pathways.

Such measures are essential to ensure a balanced, sustainable, and responsive health workforce in Bangladesh.

## Data Availability

The raw data supporting the conclusions of this article will be made available by the authors, without undue reservation.
